# Effect of conservation efforts and ecological variables on waterbird population sizes in wetlands of the Yangtze River

**DOI:** 10.1038/srep17136

**Published:** 2015-11-25

**Authors:** Yong Zhang, Qiang Jia, Herbert H. T. Prins, Lei Cao, Willem Frederik de Boer

**Affiliations:** 1 Resource Ecology Group, Wageningen University, Droevendaalsesteeg 3a, 6708PB Wageningen, The Netherlands; 2 School of Life Science, University of Science and Technology of China, 96 Jinzhai Road, Hefei 230026, Anhui, China; 3 State Key Laboratory of Urban and Regional Ecology, Research Center for Eco-Environmental Science, Chinese Academic of Sciences, 18 Shuangqing Road, Beijing, 100085, China

## Abstract

Forage quality and availability, climatic factors, and a wetland’s conservation status are expected to affect the densities of wetland birds. However, the conservation effectiveness is often poorly studied. Here, using twelve years’ census data collected from 78 wetlands in the Yangtze River floodplain, we aimed to understand the effect of these variables on five Anatidae species, and evaluate the effectiveness of the conservation measures by comparing population trends of these species among wetlands that differ in conservations status. We showed that the slope angle of a wetland and the variation thereof best explain the differences in densities of four species. We also found that the population abundances of the Anatidae species generally declined in wetlands along the Yangtze River floodplain over time, with a steeper decline in wetlands with a lower protection status, indicating that current conservation policies might deliver benefits for wintering Anatidae species in China, as population sizes of the species were buffered to some extent against decline in numbers in wetlands with a higher level protection status. We recommend several protection measures to stop the decline of these Anatidae species in wetlands along the Yangtze River floodplain, which are of great importance for the East Asian-Australasian Flyway.

Explaining and predicting animal distributions is one of the central objectives for ecologists and conservation biologists, as the species’ spatial distribution is a key variable in understanding population fluctuations^1^. Animal distribution is affected by a variety of ecological factors, such as habitat features, climatic factors and resource availability^2^. Understanding the effects of those factors on animals is still limited at a large scale where a network of wetlands that differ in suitability are included in the range that animals use. This may result in limited effectiveness of current protection measures. This issue is of great importance because the effectiveness of conservation measures along the East Asian-Australasian Flyway, especially in China, urgently needs attention because waterfowl population sizes are continuously declining^3^. Comparing population trends of a species over areas with different protection statuses can provide information with regard to the effectiveness of the protection measures. However, as long-term census data are often lacking, the effect of protection status on population trends has been poorly studied^4^ (but see work of Jesper Madsen and colleagues in Denmark^5^,^6^). Using census data of five common wintering herbivorous Anatidae species in 78 wetlands in the Yangtze River floodplain in China, we studied which factors affect Anatidae species population densities. We also analysed the species’ population trends and the effect of protection status using time series census data, available for a smaller subset of these lakes, evaluating the effectiveness of the different protection statuses in these wetlands.

Analysis of animal population trends is essential for understanding a species’ population status and, if required, for formulating protection strategies. For instance, population trends of waterbirds species in Europe indicated that loss of grassland feeding habitat negatively affected population sizes^7^,^8^. Habitat fragmentation negatively affected forest-nesting migratory birds in the United States^9^. However, an analysis which linked population trends to the effectiveness of current protection systems is generally lacking^4^, although conservation biologists and policymakers often assume to understand and address these relationships. Recently, Klein *et al.*^10^ found that conservation “paid off”, as waterbird species richness and abundance increased more rapidly in Ramsar wetlands than in unprotected wetlands in Morocco.

Many Anatidae species breed in the northern parts of Siberia, Europe and North America^11^. During the wintering period, eastern China is one of the hotspots for these migrating species in the world^12^. Eastern China supports around 1.1 million Anatidae birds and 80% of them use inland wetlands along the Yangtze River floodplain^3^,^13^. Meanwhile, these wetlands also offer food and raw materials for tens of millions of people. From 1990–2000, 30% of China’s natural wetlands have been lost due to various factors^14^. As a consequence, birds species richness in the Yangtze floodplains severely declined^3^.

In this paper, using systemic survey data from wetlands along the Yangtze River floodplain in 2004 and twelve years survey data (from 2001 to 2012) in four key wintering sites, we analysed the impact of abiotic and biotic factors on the densities of five Anatidae species to provide insight in the underlying causal factors for spatial and temporal changes in population trends, a prerequisite for effective conservation actions. Moreover, we tested the efficiency of conservation actions, and analysed whether the recent decline of Anatidae species is more severe in areas with a lower protection status compared to areas with a higher one. The species of interest were bean goose *Anser fabialis*, greater white-fronted goose *Anser albifrons*, lesser white-fronted goose *Anser erythropus*, swan goose *Anser cygnoides* and tundra swan *Cygnus columbianus bewickii*. The species selected are widely distributed in the wetlands in the Yangtze River floodplain with relatively large population sizes. Bean goose, greater and lesser white-fronted goose graze on recessional grassland, while swan goose and tundra swan mainly forage on submerged macrophytes, particularly the tubers of *Vallisneria spiralis*^15^,^16^. Hence, we expected that the grazing goose species would react to changes in *e.g.*, grass availability, but that the tuber-feeding species would not be affected by this. Instead, the tuber-feeding species were expected to be sensitive to rainfall, which changes the availability of the tubers to geese through increasing water levels.

## Results

### Effect of the ecological variables on bird density

The distribution and abundance of the studied species is shown in Supplementary information Fig. S1–S5. The majority of the variables were not significant in the zero-inflated part of the Poisson model for all species (Table 1). For the Poisson part, most variables were significantly correlated with bird density, although the effects may not be in agreement with our predictions (Table 1).

A negative individual-area relationship was found for all studied species (Table 1). Climate (temperature, rainfall) and vegetation availability (NDVI, NPP) variables had positive effects on the grazing birds. NDVI together with its square term yielded significant unimodal models for all grazing species as all these latter models had a positive main term and a negative squared term for these three species (see Supplementary Table S1 online), so a higher bird density was found at intermediate NDVI values. The effects of climate and vegetation availability on tuber-feeding birds were general negative, except for temperature that had a positive effect on tundra swan density. Slope angle variables affected bird densities differently. Slope angle was positively correlated with the grazing bird density, but negatively correlated with that of tuber-feeding birds. In contrast, the coefficient of variance of slope (SLOPECV) negatively affected grazing bird density and positively affected that of tuber-feeding birds. The spatial heterogeneity (NDVICV) negatively influenced the densities of bean goose and greater white-fronted goose, but a positive correlation was found for lesser white-fronted goose. For tuber-feeding birds, there was no effect of spatial heterogeneity on swan goose density, but a marginally significant negative effect was found on densities of tundra swan (Table 1).

According to the AICc values, the slope model was the best model explaining differences in densities of all grazing birds and tundra swans. However, the climate model best explained the density of swan goose (Table 1).

When comparing all subset models, the most parsimonious model (△AIC_C_ ≤ 2) was often the most extensive model, including most of the predictor variables (see Supplementary Table S2, S3 online). For each species, the effects of the predictor variables sometimes changed, but were generally in line with our individual predictions (see Supplementary Table S3 online). For example, not in line with our predictions, the model averaging procedure showed that both climate variables had a negative effect on the density of the greater white-fronted goose. The results showed that different mechanisms influence the bird densities of studied species simultaneously.

### Species population trends and the effect of the protection status

**Figure 1:**
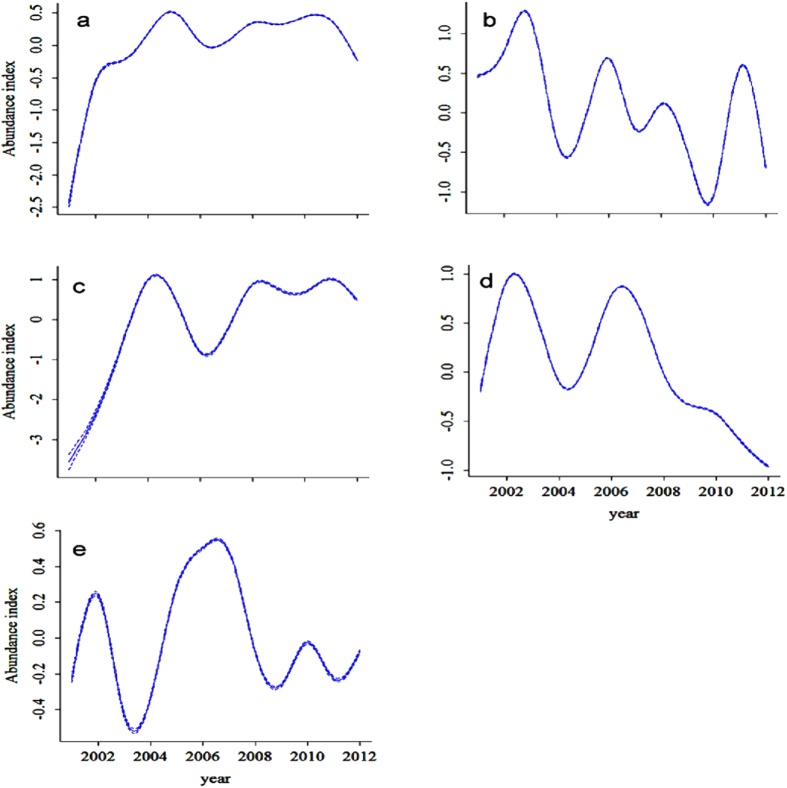
Estimated changes in population sizes of five Anatidae species from 2001 to 2012 in the Yangtze floodplain using Generalized Additive Mixed Models (GAMM). The solid line shows the population abundance index of each species and the broken lines show the 95% confidence intervals (barely visible, due to small confidence intervals). (**a**) bean goose; (**b**) greater white-fronted goose; (**c**) lesser white-fronted goose; (**d**) swan goose; (**e**) tundra swan.

The overall population abundance indices from 2001 to 2012 for the five species varied strongly according to the GAMM-results (Fig. 1). The model yielded a deviance varying from 12.5% (greater white-fronted goose) to 24.9% (bean goose). For all species, year was found to have a smoothing term significantly different from zero (Table 2). The abundance of bean goose and lesser white-fronted goose first showed an increasing trend at the beginning of the decade and then remained stable (Fig. 1a,c). The population size of the greater white-fronted goose fluctuated more and showed an overall decreasing trend (Fig. 1b). Both swan goose and tundra swan numbers decreased, especially in recent years (Fig. 1d,e).

**Figure 2:**
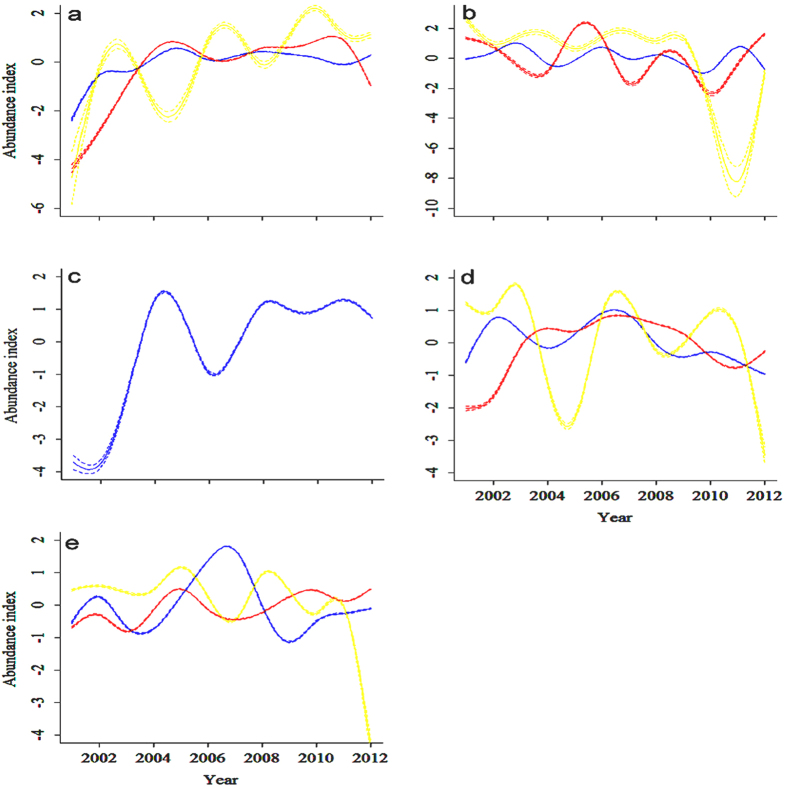
Population abundance indices of five Anatidae species from 2001 to 2012 in the 25 wetlands differing in protection status in the Yangtze floodplain using Generalized Additive Mixed Models (GAMM). Blue line: wetlands designated as national nature reserves; red line: provincial nature reserves; yellow line: county nature reserves. The solid line shows the population abundance index of each species and the broken lines show the 95% confidence intervals (barely visible, due to small confidence intervals). (**a**) bean goose; (**b**) greater white-fronted goose; (**c**) lesser white-fronted goose; (**d**) swan goose; (**e**) tundra swan. As lesser white-fronted goose was only counted in the national nature reserves, there are no population trends shown in provincial and county nature reserves for this species.

When analysing the effect of protection status, we found that bean goose and greater white-fronted goose showed a similar pattern over the three classes (i.e., national, provincial, and county nature reserve), but fluctuations were larger in reserves under a lower protection status (Fig. 2a,b). Moreover, the decreasing trends of the tuber-feeding birds in recent years in county nature reserves seemed relatively more rapid compared to the trends in national and provincial nature reserves in the floodplains of the Yangtze River (Fig. 2d,e; Table 3).

## Discussion

In this study we demonstrated that various ecological variables affected the densities of Anatidae species and the most important variables were slope and climate variables. However, these ecological variables also operated at the same time, as illustrated by the model averaging procedures. Three out of five studied species showed declining population trends with a steep decrease in recent years. Comparing the population trends among wetlands with a different protection status suggested that the largest recent declines in Anatidae species population abundances were mainly recorded from wetlands with a lower level protection status, suggesting that the current conservation policy in national nature reserves might not halt the decline in bird abundance. A larger conservation effort seems required to maintain the Anatidae population, especially for wetlands with a lower level protection status.

Our results showed that majority of the potential ecological variables significantly affected the density of Anatidae species in wetlands along the Yangtze River, although the effects sometimes were contrary to our predictions (Table 1). Slope features best explained differences in densities of all studied species except for swan goose. Partly in agreement with our hypotheses, littoral slopes had a negative effect on tuber-feeding bird density, but a positive effect on the densities of all grazing species (Table 1). Slope has a negative effect on aquatic vegetation occurrence and biomass^17^ and therefore probably negatively affected density of tuber-feeding birds. However, grazing birds on recessional grasslands may benefit from a gentle slope. For example, a gentle slope is important for an optimal habitat of Canada goose^18^. A gentle slope may also offer adequate drainage^19^, which is advantageous to littoral vegetation growth in wetland. The littoral slope in the studied wetlands was relatively flat and gentle (ranging from only 0.00 ~ 2.75°), which may explain the positive effect on grazing bird densities. However, if the range in slope angles would have been larger, we expect to find dome-shaped relationships. The coefficient of variance of these littoral slopes had a negative effect on the density of all grazing birds, but was positively correlated with that of tuber-feeding birds. Lakes with larger variation in slopes had a larger proportion of the area covered by aquatic vegetation^20^. Swan goose and tundra swan mainly forage on submerged vegetation^16^, which may explain this positive correlation.

In line with our hypothesis, mean precipitation had a positive effect on grazing bird density and a negative effect on swan goose density, but no effect was found on tundra swans. Also other studies found positive effects of precipitation on bird habitat use and density^21^. Grassland bird density increased with increasing precipitation^22^. Higher precipitation increased food availability and resulted in an increase in wintering snow goose (*Anser caerulescens*) in the USA^23^. However, a higher precipitation may also result in increasing water levels in wetlands, which decreases the food accessibility for tuber-feeding birds^24^. The found negative effect of precipitation on swan goose density is therefore expected to come from a reduction in availability of submerged vegetation. Precipitation had no effect on tundra swan density, probably because tundra swans have longer necks and hence have a higher forage availability compared to swan geese.

As predicted, temperature had a positive effect on grazing bird and tundra swan densities (Table 1). Wintering birds tend to select warmer sites to reduce the cost of thermoregulation^25^. In addition, plant primary productivity is positively correlated with temperature in grassland^26^. Unexpectedly, we found that temperature negatively influenced densities of swan goose, suggesting that densities of swan goose might be higher in higher latitude areas where temperatures are lower. However, interference competition might also play an important role in determining the distribution of herbivores^27^, and is mediated by body size^28^. Both swan goose and tundra swan are tuber-feeding birds, and when these two species forage together, interference competition may occur. Tundra swan, having a larger body size and longer necks, is expected to be the superior species, outcompeting swan goose. Another explanation for the negative effect of temperature on swan goose may be climate warming. Climate warming was a good predictor for a northward shifts in several bird species^29^,^30^,^31^. The reproductive success of waterbirds can be negatively influenced by the long distance migration from their wintering grounds to their breeding grounds^32^. As the temperatures were relatively high during the survey period, swan goose might decide to winter at higher latitude wetlands, and thereby minimize their migration distance.

Not in accordance with our predictions and former studies^33^ was that area was negatively correlated with the bird densities for all studied species, resulting in lower bird densities in lakes with larger areas available for foraging. Human activities in larger lakes may play an important role in affecting bird densities. For example, sand mining decreased food availability for birds^34^ and thereafter the density of birds in larger wetlands. It is also possible that population sizes of studied species was relatively low, resulting in lower densities in larger wetlands. For tuber-feeding species, the negative relation between area and birds densities may be partly explained by the uneven distribution of submerged aquatic vegetation among and within wetlands.

NPP had a positive effect on grazing bird densities (Table 1). NDVI yielded significant unimodal models for all three grazing bird species (see Supplementary Table S1 online). Following the forage maturation hypothesis^35^, the densities of these grazing birds first increased with increasing resource availability to a maximum level and then decreased. However, for tuber-feeding birds, NDVI and NPP had negative effects. *Carex spp.*, perennial sedges that occur in dense patches, are the dominate species of these recessional wetlands in winter. In summer, *Carex spp.* beds are flooded while the roots remain buried in the soil, which may prohibit the establishment and development of *V. spiralis,* explaining the negative correlation of NDVI and NPP on densities of tuber-feeding birds.

As expected, habitat spatial heterogeneity (NDVICV) had a negative effect on bird densities of bean goose and greater white-fronted goose and no effect on the densities of both tuber-feeding species. The positive effect on lesser white-fronted goose is probably influenced by its restricted distribution range, because the majority of lesser white-fronted goose was counted in East Dongting Lake National Reserve^36^ (Supplementary information, Fig. S3), biasing our analysis.

The results of model averaging showed that the most parsimonious model was often the most extensive model, indicating that different response variables influence bird densities at the same time (see Supplementary Table S2, S3 online). The derived correlation coefficients were generally similar between the single term models and the parsimonious multiple variables models. So, when testing several competing hypotheses, the interdependencies of those predictions should also be considered.

The recent decline of Anatidae species was more severe in areas with a lower protection status compared to areas with a higher one, which is in agreement with our expectations. Our results indicated that current conservation policies might deliver benefits for wintering Anatidae species in China, as population sizes of the studied species were buffered to some extent against a decline in numbers in wetlands with a higher level protection status. The funding that national nature reserves receive is twice as large as that of local nature reserves and the staff working in the national nature reserves have better training opportunities comparing to staff of local nature reserves^37^. Reserve staff are able to take action when more funding is received, *e.g.*, to improve wildlife protection. For example, in some national nature reserves, extra food is provided during periods when animals face food shortages. Reserves with more funding and/or a higher protection status also initiate community programs and contribute to increase the local community’s awareness, enhancing their sense of responsibility and acceptation of protection actions. In contrast, insufficient funding often leads to increased economic activities within reserves, such as the exploitation of natural resources and tourism activities^34^,^37^.

Our results, together with the studies in Europe^38^,^39^ and Africa^10^, generate a preliminary framework to evaluate the effectiveness of conservation policies. However, our analyses also had limitations as our census data were all collected from protected areas. Because of land use changes, wild birds can change their wintering site and select protected conservation areas over unprotected areas^40^. Hence, survey efforts should be broadened to cover both protected and unprotected areas in order to acquire a better understanding of the effectiveness of conservation policies.

### Application

In China, a comprehensive understanding of the spatial differences in the densities of wintering waterfowl under influence of ecological variables is still missing, reducing efficiency of protection actions. Based on our study, we suggest that hydrological regimes should be optimized to provide forage during the entire wintering period for migratory herbivorous Anatidae species. The majority of lakes along the Yangtze is connected to the Yangtze river through sluices so that management of water level heights for conservation actions is feasible. For example, through hydrological regulation, the areas of recessional grasslands for wintering birds during certain periods of the year can be increased. Water level regulation can facilitate Anatidae species grazing and regrazing by carefully timing the moment of exposure of these recessional wetlands. A sudden increase in suitable habitat will only provide preferred food in a short period, after which a “grass-sea” takes over, i.e., a wetland with a large proportion of tall and lower quality sedges. Hence, a collaborating, multidisciplinary conservation network should be built in order to formulate a scientific sound basis for protection strategies for migrating Anatidae species over a network of wetlands.

To better evaluate the effectiveness of the protection actions, a systematic annual waterbirds survey should be carried out both in protected and unprotected areas by Chinese government departments such as the state forest bureaus in collaboration with scientists, and the data should be freely available. For example, the North American Breeding Bird Survey (BBS) was initiated in 1966 and the survey is conducted every year. The main objective is to track the status and trends of North American bird populations and data can be retrieved freely from a public website. In the Netherlands, SOVON started in 1973, carrying out standardised annual national bird surveys. We strongly advocate that China starts an annual wintering birds survey, offering a basis for current and future conservation work.

Furthermore, we suggest that it is time to involve birdwatchers and volunteers in China’s conservation network. Larger survey projects can strongly benefit from contributions from birdwatchers and volunteers. Birdwatchers and volunteers are often highly motivated and skilled, and can contribute to surveys. For example, thousands of volunteer birdwatchers participated in the Breeding Bird Survey in the UK. Nowadays, the number of birdwatchers is increasing in China and they can contribute to the necessary bird surveys.

Finally, we claim that nature reserves with a lower protection status should also be given more attention in terms of investment, local community education and research efforts. Some lower protection status wetlands, such as the Anhui Anqing Yangtze Riverine Provincial Nature Reserve, could be upgraded to a national nature reserve to increase the conservation efforts in this important wetland. Moreover, even the national nature reserves are apparently not sufficient to stop the decline of the Anatidae birds, and thus additional measures are required. We therefore call for an in-depth investigation into the decline of Anatidae species in the East Asian-Australasian Flyway, as contrasted to the successes of the American and European counterparts.

## Methods

### Census data

Data from the studied five Anatidae species was obtained from the middle-lower Yangtze River floodplain survey carried out in February 2004, the first comprehensive survey in this area^41^. All selected species are herbivorous birds wintering in the wetlands in the Yangtze River floodplain^13^. We only selected data from lakes, whereas estuaries and shoals were excluded from the analysis. The whole dataset included 78 lakes over 5 provinces (see Supplementary Table S4 online). Another dataset was obtained from a systematic survey in four nature reserves (Poyang Hu, Dongting Hu, Shengjin Hu and Anqing lakes) of waterbirds in the winters from 2000/1 through 2011/12 (see Supplementary Table S5 online). The “look-see” counting method is commonly used to count waterbirds^42^ and was used for all surveys. The“look-see” counting method required the observers to be familiar with the species involved and their habitat-preferences^42^. Multiple methods were used to access the wetlands and birds, but in most cases cars were employed to reach the target areas as close as possible and then the observers proceeded on foot. Most Anatidae often gather in large visible flocks during the wintering season, making them easy to locate and count^43^. The surveys were conducted by staff of the nature reserve and by the authors using the same survey methods; detailed survey methods are described in Barter *et al.*^41^.

### Variables

#### Lake land and water area

Previous studies have pointed out that habitat area positively affects bird density^33^,^44^. Grazing Anatidae species wintering in the Yangtze River floodplain mainly feed on recessional grasslands. The size of the grassland that is exposed, and hence available to grazing birds for foraging, increases with decreasing lake water levels and thereby affects the density of these birds. We related the density of tuber-feeding birds to lake water area as they mainly forage on submerged *V. spiralis* tubers^15^,^16^. For tuber-feeding birds a similar positive relationship was expected, although the size of the lake area is positively correlated to height of the water level, and therefore maybe negatively with the accessibility of the tubers^24^. We measured lake land and water area of the studied 78 wetlands during the wintering survey in 2004 using satellite images. The data description is shown in Table 4, with detailed methods available in the Supplementary information Appendix S1.

#### Littoral slopes

Vegetation growth is often affected by lake morphology such as littoral slopes. Littoral slopes negatively affect vegetation occurrence and biomass^17^ and thereby also the densities of herbivorous Anatidae species^18^. A gentle slope is therefore more suitable for vegetation development in wetlands^20^,^45^. Thus, we predict that Anatidae species densities will be negatively correlated with the mean littoral slope angle. In addition, variation of the wetlands’ littoral slope angles may also affect vegetation growth, with highest growth rates and biomass often found on gentle slopes^46^. We hence predicted a negative effect of the coefficient of variation (CV) of littoral slope angles on bird densities. We calculated the average and CV of littoral slope angles of each lake using Shuttle Radar Topography Mission (SRTM) digital elevation data from February 2000 (Table 4) as topography changes were negligible from 2000 to 2004.

#### Climate data

Weather conditions can affect bird distribution and density through changing temperatures and precipitation^47^. The abundance of wintering birds normally decreased with decreasing temperatures in winter^47^ (but see Ridgill & Fox^48^). Root^49^ suggested that this could be explained by the species’ energy expenditure. Moreover, plant primary productivity is positively correlated with temperature. We therefore expected that bird densities will be positively correlated with temperature. Precipitation positively affects plant primary productivity^26^, but these effects often have a time lag in influencing vegetation availability of about a month^50^. We therefore also related mean January (i.e. the previous month for the surveys) precipitation to the densities of grazing birds, expecting a positive effect. However, lake water level increases with increasing precipitation, and the food accessibility for tuber-feeding birds, which is dependent on water depth and the bird’s neck length, therefore decreases^24^. Hence, we predicted that densities of tuber-feeding birds will be negatively correlated with mean precipitation. Monthly mean air temperatures and precipitation were obtained from the China Meteorological Administration (Table 4).

#### Normalized Difference Vegetation Index (NDVI) and Net Primary Productivity (NPP)

Forage quantity is an important variable in determining animal distribution^51^,^52^. So NDVI and NPP were used as predictors in the density analyses of grazing birds. As functional response curves suggest that animal densities are correlated to forage biomass through a unimodal relationship^52^, we hence included its square terms, NDVI^2^ and NPP^2^, in the analysis. For tuber-feeding birds, we expected that NDVI and NPP have no effect on bird density as these species mainly forage on tubers but not on grass. We calculated the mean NPP (Table 4) per lake, and the mean NDVI for only recessional grasslands per lake using the satellite images (see Supplementary Table S6 online). The detailed image processing methods are available in the Supplementary information, Appendix S1.

#### Habitat heterogeneity

Studies showed that habitat heterogeneity can decrease foraging efficiency of grazers by increasing searching and handling times^53^. Intake rates of herbivores are generally lower while feeding on heterogeneous swards compared to homogenous swards, such as shown for several overwintering waterbird species (*e.g.*, *Anser* spp., *Anas* spp.)^52^ and habitat heterogeneity is therefore expected to affect grazing bird density negatively, but not affect tuber-feeding bird density. We calculated the CV of NDVI from the different pixels in the same period (see above) as an index of the spatial heterogeneity in forage availability at these recessional grasslands for each lake, expecting a negative correlation with bird density (Table 4).

#### Protection status

Establishing protected area is a cornerstone for maintaining the global biodiversity^54^. Birds species benefitted from various conservation measures in Europe^38^,^39^. Moreover, waterbirds increased more rapidly in Ramsar-designated wetlands in Morocco compared to unprotected wetlands^10^.

China’s protected area system includes national, provincial, city and county nature reserves, with some wetlands designated as Ramsar sites. Provincial, city and county nature reserves are often poorly managed because of reduced funding compared to national nature reserves^37^. As city nature reserves were not available in our second dataset, we therefore categorized our research lakes into national, provincial, and county nature reserve according to the list of China’s nature reserves (State Ministry of Environmental Protection 2012). We predicted that national reserves would have a stronger positive effect on population trends compared to the wetlands with a lower protection status.

#### Statistical analysis

Following the above reasoning we formulated a set of working hypotheses. Model I represents the effect of habitat area on the bird density of Anatidae species (Individual-area relationship). Model II, III, IV, V represent effect of climate, vegetation availability, slope and spatial heterogeneity respectively (Table 5).

Count data often include many zero observations. Poisson regression can be used to model the relationship between species abundance and environmental variables, but zero-inflated Poisson models often perform better than Poisson models or zero-inflated negative binomial models^55^. Hence, a zero-inflated Poisson model was applied to analyse the effects of different ecological variables on bird densities. A zero-inflated Poisson model includes two parts: a Poisson model and a zero-inflated model. The zero-inflated part provides insight on variables influencing the species’ presence/absence while the Poisson part provides insight on the variables affecting the species’ density. We performed a zero-inflated Poisson regression analysis for each of the hypotheses. The Akaike Information Criterion (AIC), adjusted for small sample sizes (AIC_C_), was used to rank the competing models. Before fitting the zero-inflated Poisson models, we assessed the multi-collinearity by examining the Variance Inflation Factor (VIF) of the candidate variables, by including all candidate variables as independent variables in a regression model with animal density as response variable. VIF values of all variables were less than 4 (see Supplementary Table S7 online), indicating that there was no multi-collinearity problem^56^.

Furthermore, different mechanisms may influence the density of each species at the same time, but distinguishing their independent effect is a challenging task^57^. Hence, zero-inflated Poisson models were also used to test for the combined and independent influence of the predictor variables on the densities of each of the species. All possible subset models were ranked according to △AICc and Akaike weights (ωi) were calculated to estimate the likelihood of each model^58^. Model averaging was used to obtain parameter estimates for these variables. The model averaging calculation was done on the most parsimonious models using a cut-off △AIC_C_ ≤ 2^58^.

To analyse population trends for each of the five waterbird species, a Generalized Additive Mixed Model (GAMM) was applied using the time series survey data (2001–2012) from 25 wetlands in the four nature reserves where birds counts were carried out annually, with province as random factor. The GAMM model accommodates for smooth, nonlinear changes over time in population size^59^. In the model (Eq. 1), y_ij_ is the expected bird count at site i and year j. The expected count therefore depends on the site effect a_i_ and the smoother s(j). The analysis was done in two parts: we first analysed the overall population trends of each species in these wetlands. Then another GAMM was applied for each species but separately for the wetlands with a different protection status (national, provincial, and county). We used a GAMM with a Poisson distribution and a log link function (Eq. 2).


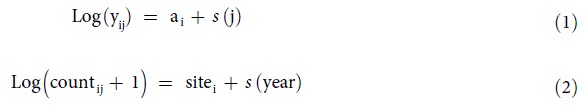


Spatial autocorrelation is a potential problem when analysing ecological data and should be properly accounted for. We therefore explored whether there was spatial autocorrelation in birds abundances over different wetlands by calculating the Moran’s I index of the residuals for each species. We found little evidence for spatial autocorrelation of studied species (all |Moran’s I| < 0.05) which suggested that spatial autocorrelation was not a point of concern in our analysis. All statistical analyses were conducted in R 2.13.0^60^ with the package pscl, MuMIn, mgcv and ape.

## Additional Information

**How to cite this article**: Zhang, Y. *et al.* Effect of conservation efforts and ecological variables on waterbird population sizes in wetlands of the Yangtze River. *Sci. Rep.* **5**, 17136; doi: 10.1038/srep17136 (2015).

## Supplementary Material

Supplementary Information

## Figures and Tables

**Table 1:** Predicted (H_0_) and observed effects (+: positive effect; −: negative effect; NS: no effect) of different variables on the bird density of five study species tested for each competing hypotheses using a zero-inflated Poisson regression model (b = regression coefficient, se = standard error, z = calculated z-value, p = significance, AIC_c_ = sample size corrected Akaike Information Criterion).

Species	Model	Variables	H0	Poisson model				zero-inflated model				
				b	se	z	p	b	se	z	p	AIC_C_
BG	Model I	LA	+	−0.007	0.001	−11.3	<0.001	−0.023	0.012	−1.913	0.056	8218
	Model II	TEMP	+	1.271	0.038	33.53	<0.001	0.161	0.397	0.406	0.685	6913
		MP	+	0.085	0.003	26.69	<0.001	−0.060	0.033	−1.848	0.065	
	Model III	NDVI	+	3.817	0.313	12.190	<0.001	−0.990	4.498	−0.220	0.826	8228
		NPP	+	0.007	0.001	5.311	<0.001	−0.041	0.018	−2.230	0.026	
	Model IV†	SLOPE	−	0.951	0.031	30.33	<0.001	−0.524	0.498	−1.053	0.293	6554
		SLOPECV	−	−3.008	0.095	−31.59	<0.001	−1.428	1.125	−1.269	0.204	
	Model V	NDVICV	−	−4.610	0.277	−16.67	<0.001	−2.003	4.201	−0.477	0.633	8124
GWFG	Model I	LA	+	−0.007	0.002	−4.583	<0.001	0.008	0.010	0.792	0.429	4157
	Model II	TEMP	+	0.720	0.053	13.517	<0.001	−0.483	0.339	−1.424	0.154	3922
		MP	+	0.016	0.004	4.156	<0.001	0.091	0.038	2.379	0.017	
	Model III	NDVI	+	12.690	0.551	23.020	<0.001	−4.038	4.554	−0.887	0.375	3611
		NPP	+	0.011	0.002	6.244	<0.001	−0.024	0.018	−1.297	0.195	
	Model IV†	SLOPE	−	0.788	0.039	20.04	<0.001	0.218	0.519	0.420	0.675	3453
		SLOPECV	−	−3.124	0.164	−19.04	<0.001	−0.067	1.079	−0.062	0.950	
	Model V	NDVICV	−	−4.999	0.568	−8.801	<0.001	−0.317	4.331	−0.073	0.942	4099
LWFG	Model I	LA	+	−0.008	0.001	−7.065	<0.001	−0.013	0.008	−1.558	0.119	2316
	Model II	TEMP	+	2.907	0.189	15.40	<0.001	−1.421	0.998	−1.424	0.154	1435
		MP	+	0.201	0.017	11.98	<0.001	−0.041	0.057	−0.710	0.478	
	Model III	NDVI	+	0.543	0.820	0.662	0.508	−18.11	6.676	−2.713	0.007	1608
		NPP	+	0.080	0.004	22.023	<0.001	0.011	0.028	0.400	0.689	
	Model IV†	SLOPE	−	2.261	0.074	30.51	<0.001	0.184	0.723	0.254	0.800	630
		SLOPECV	−	−2.431	0.200	−12.17	<0.001	−0.264	1.465	−0.180	0.857	
	Model V	NDVICV	−	2.381	0.655	3.633	<0.001	12.719	6.744	1.886	0.059	2200
SG	Model I	WA	+	−0.012	0.001	−17.37	<0.001	−0.006	0.004	−1.445	0.148	5129
	Model II†	TEMP	+	−3.659	0.085	−42.97	<0.001	0.403	0.326	1.237	0.216	1563
		MP	−	−0.134	0.004	−34.90	<0.001	−0.080	0.036	−2.228	0.026	
	Model III	NDVI	NS	−3.617	0.382	−9.461	<0.001	4.368	5.557	0.786	0.432	5613
		NPP	NS	0.002	0.002	0.870	0.384	−0.031	0.021	−1.474	0.140	
	Model IV	SLOPE	−	−3.184	0.131	−24.37	<0.001	−0.485	0.670	−0.723	0.470	3591
		SLOPECV	−	5.183	0.146	35.53	<0.001	−1.590	1.270	−1.253	0.210	
	Model V	NDVICV	NS	0.185	0.287	0.645	0.519	1.461	5.023	0.291	0.771	5708
TS	Model I	WA	+	−0.024	0.001	−20.58	<0.001	−0.010	0.008	−1.294	0.196	5027
	Model II	TEMP	+	0.571	0.041	13.807	<0.001	0.207	0.272	0.761	0.446	5546
		MP	−	−0.002	0.003	−0.627	0.531	−0.015	0.029	−0.514	0.607	
	Model III	NDVI	NS	−4.993	0.436	−11.44	<0.001	2.869	4.724	0.607	0.544	5370
		NPP	NS	−0.030	0.002	−18.94	<0.001	−0.037	0.019	−1.981	0.048	
	Model IV†	SLOPE	−	−2.057	0.076	−27.12	<0.001	−0.754	0.527	−1.432	0.152	4782
		SLOPECV	−	2.359	0.118	19.96	<0.001	−0.101	1.094	−0.092	0.926	
	Model V	NDVICV	NS	−0.722	0.364	−1.983	0.047	2.099	4.368	0.481	0.631	5832

BG: bean goose; GWFG: greater white-fronted goose; LWFG: lesser white-fronted goose; SG: swan goose; TS: tundra swan. For variable abbreviation see Table 4.

^†^best competing model.

**Table 2:** Results of the Generalized Additive Mixed Model (GAMM) analysing the overall changes in population sizes of five Anatidae species from 2001 to 2012 in wetlands of the Yangtze floodplain.

Species	Smooth terms					Explanatory variables
	UBRE	Deviance explained (%)	edf	χ^2^	p	site
BG	5321	24.9	8.945	40391	<0.001	<0.001
GWFG	5574	12.5	8.976	97537	<0.001	<0.001
LWFG	2155	15.6	8.973	33465	<0.001	<0.001
SG	7137	20.7	8.924	223695	<0.001	<0.001
TS	4615	12.7	8.938	49992	<0.001	<0.001

BG: bean goose; GWFG: greater white-fronted goose; LWFG: lesser white-fronted goose; SG: swan goose; TS: tundra swan. UBRE: Un-Biased Risk Estimator; edf: effective degrees of freedom (n = 78).

**Table 3:** Results of the Generalized Additive Model (GAMM) analysing the changes in population sizes of five Anatidae species from 2001 to 2012 in 25 wetlands with different protection statuses in the Yangtze floodplain.

	Species	Smooth terms					Explanatory variable
		UBRE	Deviance explained (%)	edf	χ^2^	p	site
National nature reserve (n = 6)	BG	6735	22.2	8.976	20598	<0.001	<0.001
	GWFG	7570	43.7	8.992	121895	<0.001	<0.001
	LWFG	4488	17.5	8.964	22898	<0.001	<0.001
	SG	9486	51.5	8.978	174450	<0.001	<0.001
	TS	3849	54.2	8.980	115464	<0.001	<0.001
Provincial nature reserve (n = 11)	BG	6655	13.8	8.922	50873	<0.001	<0.001
	GWFG	354	45.5	8.987	11794	<0.001	<0.001
	LWFG	39	46.1	8.888	805	<0.001	<0.001
	SG	3352	11.5	8.959	33164	<0.001	<0.001
	TS	4388	10.7	8.971	20799	<0.001	<0.001
County nature reserve (n = 8)	BG	286	22.9	8.932	4616	<0.001	<0.001
	GWFG	339	24.9	8.988	5505	<0.001	<0.001
	LWFG	22	58.3	6.746	253	<0.001	<0.001
	SG	957	24.3	8.983	13950	<0.001	<0.001
	TS	2725	11.1	8.979	13923	<0.001	<0.001

BG: bean goose; GWFG: greater white-fronted goose; LWFG: lesser white -fronted goose; SG: swan goose; TS: tundra swan. UBRE: Un-Biased Risk Estimator; edf: effective degrees of freedom.

**Table 4:** Potential predictor variables, abbreviations, data sources and resolutions used to analyse differences in species abundance in wetlands of the Yangtze River floodplain.

Variables	Abbreviation	Unit	Range	Source	Resolution
Lake land area	LA	km^2^	0.20 ~ 216.04	landsat TM/ETM+	30 m
Water area	WA	km^2^	0.13 ~ 1612.16	landsat TM/ETM+	30 m
February mean air temperature	TEMP	°C	7.30 ~ 11.20	http://www.cma.gov.cn/2011qxfw/2011qsjgx/	0.5° × 0.5°
Mean January precipitation	MP	mm	3.70 ~ 158.60	http://www.cma.gov.cn/2011qxfw/2011qsjgx/	0.5° × 0.5°
Littoral slopes	SLOPE	°	0.00 ~ 2.75	http://srtm.csi.cgiar.org	90 m
Coefficient of variance of littoral slopes	SLOPECV	no unit	0.00 ~ 1.49	http://srtm.csi.cgiar.org	90 m
Normalized difference vegetation index	NDVI	no unit	0.20 ~ 0.43	landsat TM/ETM+	30 m
Net primary productivity	NPP	g/m^2^ month^−1^	52.00 ~ 98.60	http://neo.sci.gsfc.nasa.gov/	0.1° × 0.1°
Habitat heterogeneity	NDVICV	no unit	0.08 ~ 0.35	landsat TM/ETM+	30 m

**Table 5:** Theoretical models expected to affect the densities of Anatidae species in wetlands.

Theoretical model	LA/WA	TEMP	MP	SLOPE	SLOPECV	NDVI	NPP	NDVICV
Model I								
Individual-area relationship	X							
Model II								
Climate		X	X					
Model III								
Slope				X	X			
Model IV								
Vegetation availability						X	X	
Model V								
Spatial heterogeneity								X
