# Supplementary material for: Effect of conservation efforts and ecological variables on waterbird population sizes in wetlands of the Yangtze River
Source: Sci Rep. 2015 Nov 25;5:17136. doi: 10.1038/srep17136 (PMC4658538; doi:10.1038/srep17136)
Supplement: Supplementary Information [file srep17136-s1.pdf]

**Supplementary information**

# Effect of conservation efforts and ecological variables on waterbird population sizes in wetlands of the Yangtze River

Yong Zhang[1,*], Qiang Jia[2], Herbert H.T. Prins[1], Lei Cao[3,*], and Willem Frederik de Boer[1]

1 Resource Ecology Group, Wageningen University, Droevendaalsesteeg 3a, 6708PB Wageningen, The Netherlands

2 School of Life Science, University of Science and Technology of China, 96 Jinzhai Road, Hefei 230026, Anhui, China

3 State Key Laboratory of Urban and Regional Ecology, Research Center for Eco-Environmental Science, Chinese Academic of Sciences, 18 Shuangqing Road, Beijing, 100085, China

*Correspondence to Yong Zhang E-mail: zyong@mail.ustc.edu.cn or Lei Cao E-mail: leicao@rcees.ac.cn

**Figure S1:** Distribution and abundance of bean goose in the Yangtze Flood Plain in 2004, with different colours for different classes of population abundances. The break values were set to 0, 1, 100, 800 (1% of estimated population size) and 4000 (5% of estimated population size). (The figure was created by Y. Zhang in ArcGIS 10.0 software, http://www.esri.com/).

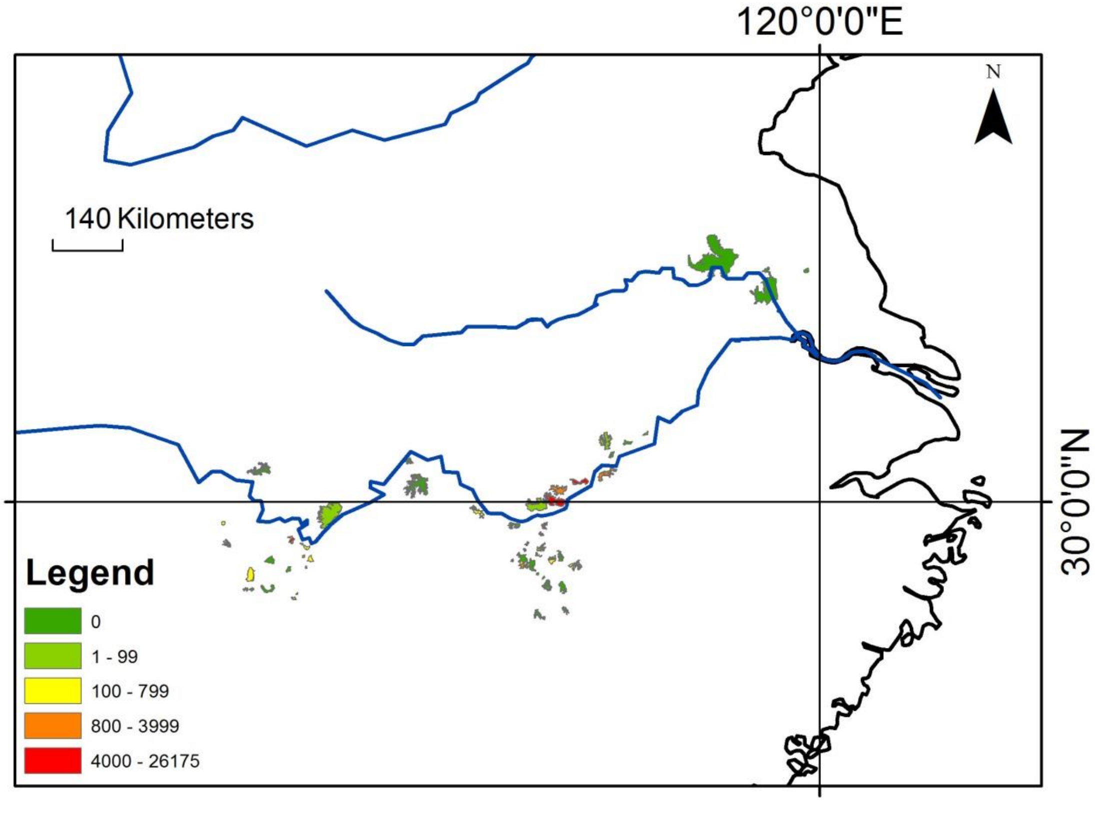

**Figure S2:** Distribution and abundance of greater white-fronted goose in the Yangtze Flood Plain in 2004, with different colours for different classes of population abundances. The break values were set to 0, 1, 100, 1800 (1% of estimated population size) and 9000 (5% of estimated population size). (The figure was created by Y. Zhang in ArcGIS 10.0 software, http://www.esri.com/).

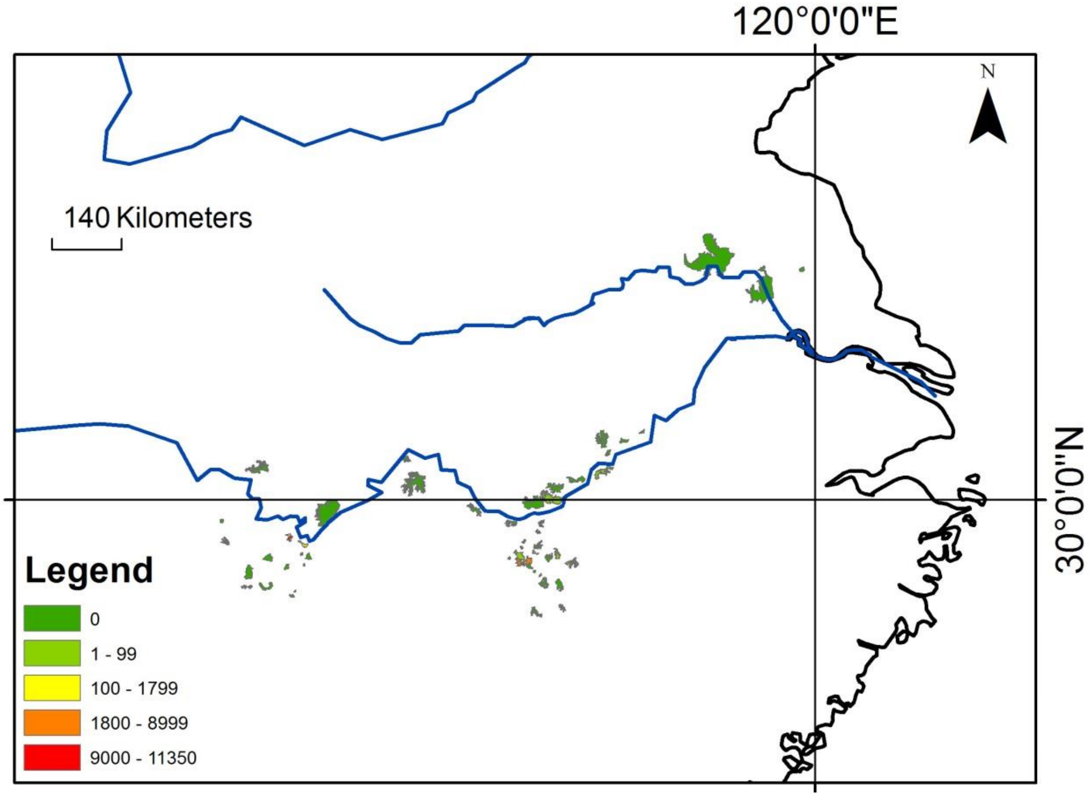

**Figure S3:** Distribution and abundance of lesser white-fronted goose in the Yangtze Flood Plain in 2004, with different colours for different classes of population abundances. The break values were set to 0, 1, 100, 200 (1% of estimated population size) and 1000 (5% of estimated population size). (The figure was created by Y. Zhang in ArcGIS 10.0 software, http://www.esri.com/).

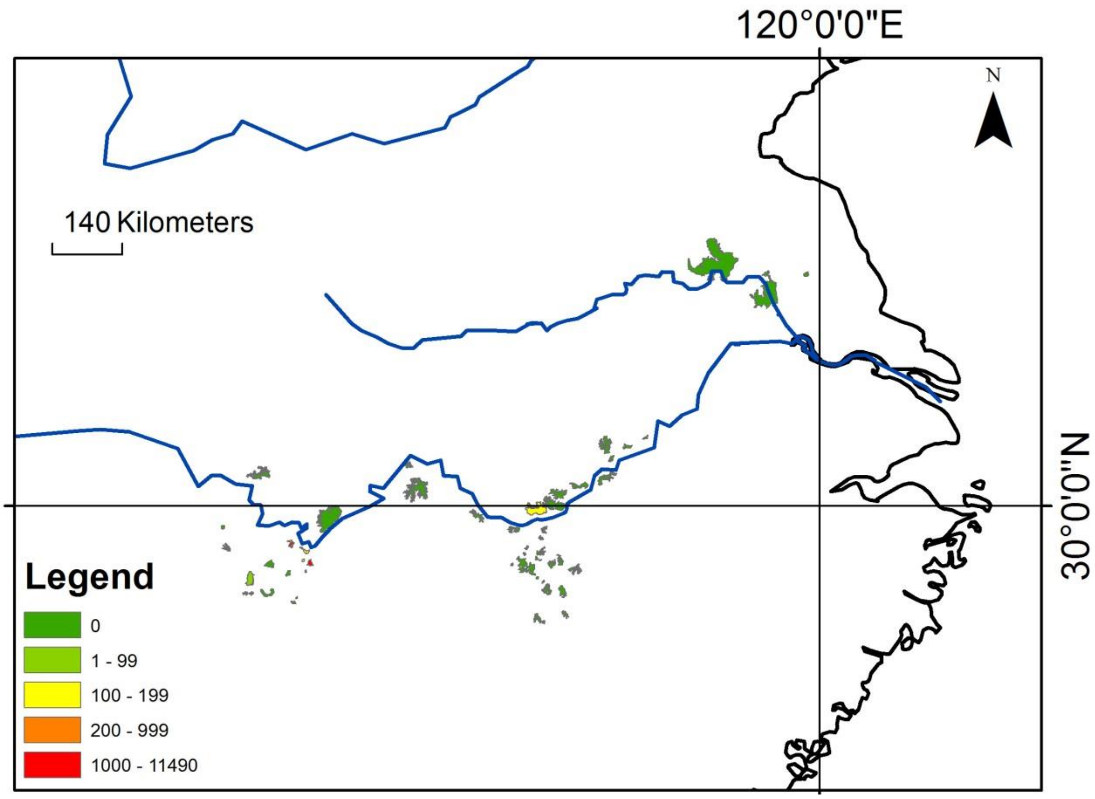

**Figure S4:** Distribution and abundance of swan goose in the Yangtze Flood Plain in 2004, with different colours for different classes of population abundances. The break values were set to 0, 1, 100, 800 (1% of estimated population size) and 4000 (5% of estimated population size). (The figure was created by Y. Zhang in ArcGIS 10.0 software, http://www.esri.com/).

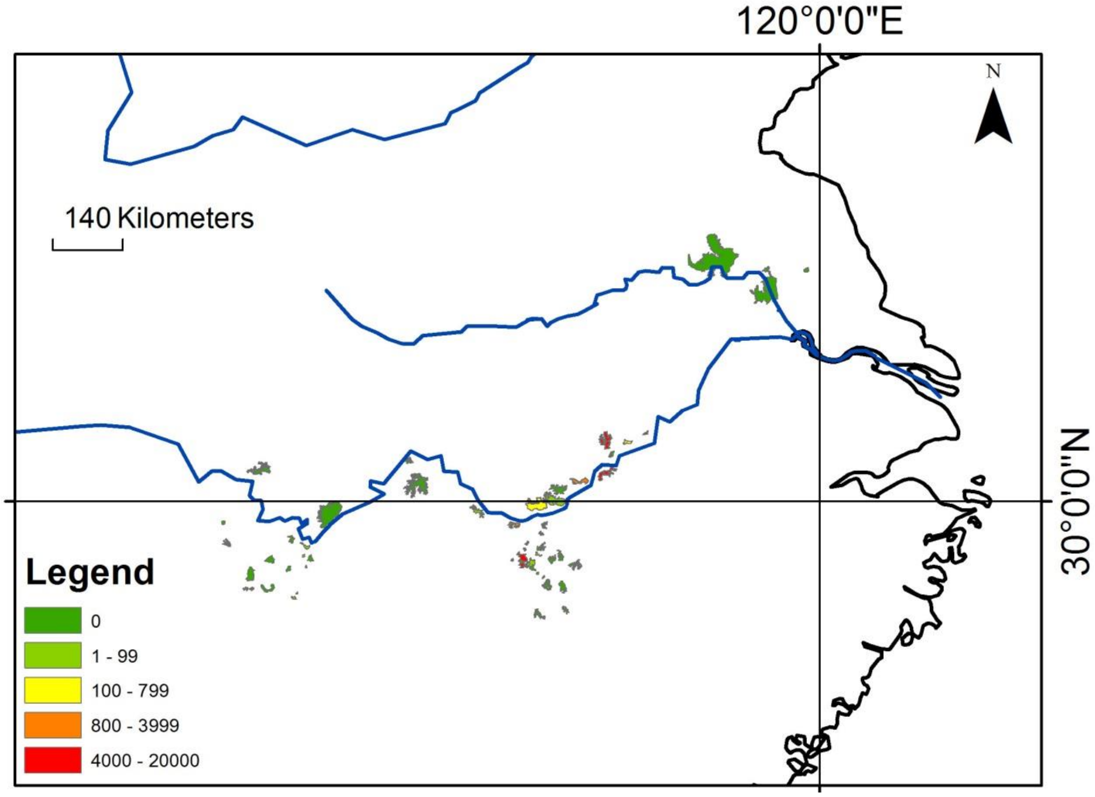

**Figure S5:** Distribution and abundance of tundra swan in the Yangtze Flood Plain in 2004, with different colours for different classes of population abundances. The break values were set to 0, 1, 100 and 920 (1% of estimated population size). (The figure was created by Y. Zhang in ArcGIS 10.0 software, http://www.esri.com/).

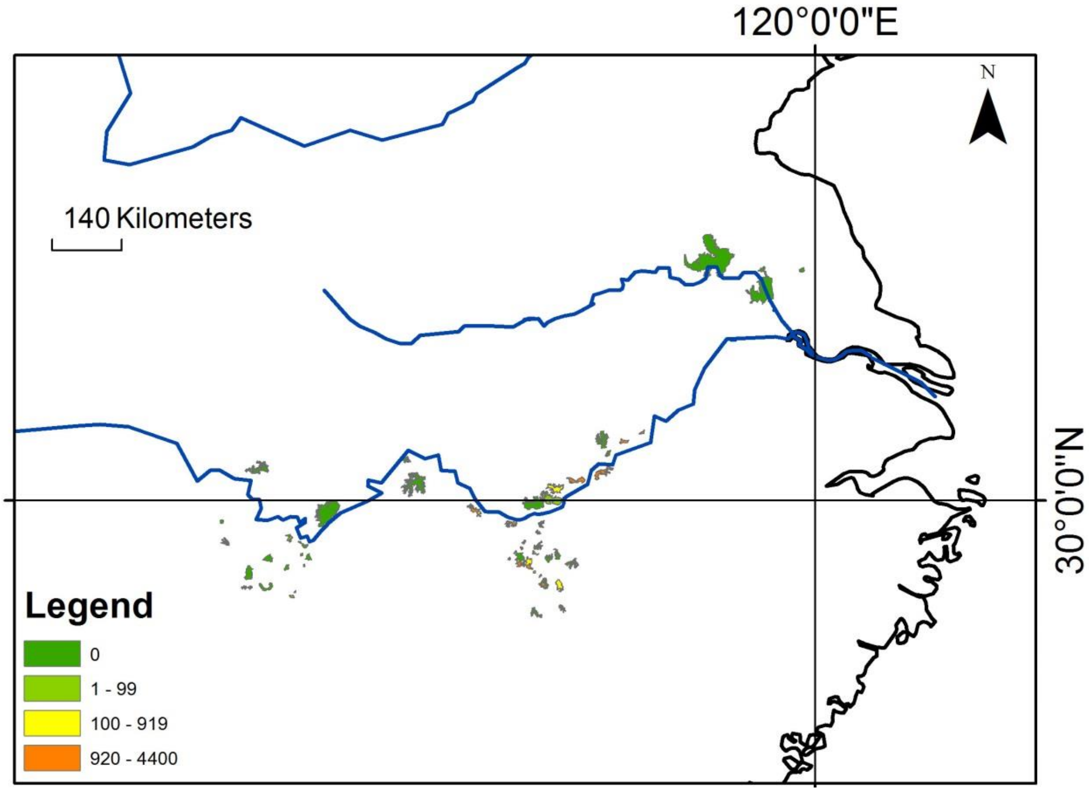

Table S1: Effect of NDVI together with its square term on the species density of grazing birds. BG: bean goose; GWFG: greater white-fronted goose; LWFG: lesser white-fronted goose; SG. For variable abbreviation see Table 4.

| Species | Variables | Poisson model | | | | zero-inflated model | | | |
|---|---|---|---|---|---|---|---|---|---|
| | | b | se | z | p | b | se | z | p |
| BG | $NDVI^2$ | -85.946 | 6.422 | -13.382 | <0.001 | 23.769 | 77.058 | 0.308 | 0.758 |
| | NDVI | 58.216 | 4.070 | 14.304 | <0.001 | -14.929 | 46.786 | -0.319 | 0.750 |
| GWFG | $NDVI^2$ | -330.076 | 17.814 | -18.53 | <0.001 | 230.15 | 110.03 | 2.092 | 0.037 |
| | NDVI | 218.218 | 11.185 | 19.51 | <0.001 | -142.99 | 66.93 | -2.136 | 0.033 |
| LWFG | $NDVI^2$ | -447.769 | 27.652 | -16.19 | <0.001 | -56.361 | 131.270 | -0.429 | 0.668 |
| | NDVI | 301.722 | 18.491 | 16.32 | <0.001 | 19.395 | 84.254 | 0.230 | 0.818 |

**Table S2:** Results from the zero-inflated Poisson model aiming to explain the effects of different variables on bird densities (AICc = sample size corrected Akaike Information Criterion. K = number of explanatory parameters in model; $\Delta AIC_C$ = AICc(i) - AICc(min); ωi = Akaike weights). BG: bean goose; GWFG: greater white-fronted goose; LWFG: lesser white-fronted goose; SG: swan goose; TS: tundra swan. For variable abbreviation see Table 4.

| Species | Model | LA/WA | TEMP | MP | SLOPE | SLOPECV | NDVI | NPP | NDVICV | k | logLik | $AIC_C$ | $\Delta AIC_C$ | ωi |
|---|---|---|---|---|---|---|---|---|---|---|---|---|---|---|
| BG | 1† | X | X | X | X | X | | X | X | 16 | -2792.3 | 5625.6 | 0.00 | 0.761 |
| | 2 | X | X | X | X | X | X | X | X | 18 | -2790.3 | 5628.2 | 2.62 | 0.205 |
| GWFG | 1† | X | | X | X | X | X | X | X | 16 | -1096.7 | 2234.3 | 0.00 | 0.633 |
| | 2† | X | X | X | X | X | X | X | X | 18 | -1094.1 | 2235.7 | 1.39 | 0.316 |
| | 3 | X | X | X | X | X | X | | X | 16 | -1099.2 | 2239.4 | 5.07 | 0.050 |
| LWFG | 1† | X | X | | X | | X | | X | 12 | -82.5 | 193.9 | 0.00 | 0.670 |
| | 2 | X | X | | X | | X | X | X | 14 | -80.9 | 196.5 | 2.62 | 0.181 |
| SG | 1† | X | X | X | X | X | X | X | X | 18 | -298.1 | 643.8 | 0.00 | 0.816 |
| | 2 | X | X | X | X | X | X | | X | 16 | -302.9 | 646.7 | 2.97 | 0.184 |
| TS | 1† | X | X | X | X | X | X | X | X | 18 | -1446.6 | 2940.8 | 0.00 | 1.000 |
| | 2 | X | X | X | X | X | X | | X | 16 | -1464.3 | 2969.5 | 28.69 | 0.000 |

†: best competing model(s)

Table S3: Predicted ($H_0$) and observed effects (+: positive effect; -: negative effect; NS: no effect) of different variables on bird density of five study species using a zero-inflated Poisson regression model based on AICc model selection with model averaging (b = regression coefficient, se = standard error, 95% CI = 95% confidence interval). BG: bean goose; GWFG: greater white-fronted goose; LWFG: lesser white-fronted goose; SG: swan goose; TS: tundra swan. For variable abbreviation see Table 4.

| Species | Variables | H0 | Poisson model | | | Zero-inflated model | | |
|---|---|---|---|---|---|---|---|---|
| | | | b | se | 95% CI | b | se | 95% CI |
| BG | LA | + | -0.006 | 0.001 | -0.007, -0.004 | -0.017 | 0.012 | -0.041, 0.007 |
| | TEMP | + | 0.606 | 0.047 | 0.514, 0.698 | -0.328 | 0.437 | -1.185, 0.529 |
| | MP | + | 0.074 | 0.003 | 0.067, 0.081 | -0.053 | 0.036 | -0.123, 0.018 |
| | SLOPE | - | 0.906 | 0.040 | 0.828, 0.984 | -0.324 | 0.522 | -1.347, 0.699 |
| | SLOPECV | - | -2.588 | 0.121 | -2.826, -2.350 | -1.385 | 1.263 | -3.861, 1.091 |
| | NDVI | + | | | | | | |
| | NPP | + | -0.006 | 0.002 | -0.010, -0.002 | -0.041 | 0.021 | -0.081, -0.000 |
| | NDVICV | - | -2.721 | 0.341 | -3.389, -2.053 | 2.513 | 4.855 | -7.002, 12.028 |
| GWFG | LA | + | -0.045 | 0.004 | -0.053, -0.039 | -0.011 | 0.020 | -0.053, 0.026 |
| | TEMP | + | -0.065 | 0.108 | -0.384, -0.009 | -0.152 | 0.316 | -1.242, 0.327 |
| | MP | + | -0.075 | 0.007 | -0.084, -0.056 | 0.070 | 0.042 | -0.005, 0.166 |
| | SLOPE | - | 1.346 | 0.058 | 1.269, 1.492 | 0.356 | 0.601 | -0.816, 1.515 |
| | SLOPECV | - | -5.125 | 0.233 | -5.641, -4.730 | -1.365 | 1.489 | -4.303, 1.697 |
| | NDVI | + | 28.45 | 0.997 | 26.71, 30.63 | 0.213 | 5.548 | -9.985, 11.816 |
| | NPP | + | 0.010 | 0.003 | 0.003, 0.014 | -0.030 | 0.022 | -0.070, 0.014 |
| | NDVICV | - | 14.467 | 0.904 | 13.00, 16.56 | 3.194 | 6.074 | -9.731, 13.678 |
| LWFG | LA | + | -0.012 | 0.002 | -0.017, -0.006 | -0.050 | 0.029 | -0.106, 0.006 |
| | TEMP | + | 3.970 | 0.301 | 3.380, 4.561 | -1.163 | 1.613 | -4.325, 2.000 |
| | MP | + | | | | | | |
| | SLOPE | - | 1.742 | 0.073 | 1.599, 1.886 | 0.488 | 0.726 | -0.934, 1.911 |
| | SLOPECV | - | | | | | | |
| | NDVI | + | 17.99 | 1.447 | 15.155, 20.825 | -10.298 | 8.262 | -26.491, 5.894 |
| | NPP | + | | | | | | |
| | NDVICV | - | 29.01 | 2.047 | 25.00, 33.02 | 23.591 | 12.308 | -0.533, 47.715 |
| SG | WA | + | -0.017 | 0.001 | -0.018, -0.014 | -0.066 | 0.027 | -0.119, -0.013 |
| | TEMP | + | -2.113 | 0.120 | -2.348, -1.877 | 0.183 | 0.588 | -0.970, 1.336 |
| | MP | - | -0.145 | 0.005 | -0.154, -0.136 | -0.066 | 0.052 | -0.168, 0.037 |
| | SLOPE | - | -2.856 | 0.181 | -3.210, -2.501 | -1.245 | 1.350 | -3.890, 1.400 |
| | SLOPECV | - | 6.940 | 0.509 | 5.942, 7.939 | 2.279 | 2.300 | -2.228, 6.786 |
| | NDVI | NS | 5.763 | 0.738 | 4.318, 7.209 | 4.325 | 8.028 | -11.410, 20.060 |
| | NPP | NS | -0.008 | 0.004 | -0.017, 0.001 | -0.085 | 0.036 | -0.156, -0.014 |
| | NDVICV | NS | -6.713 | 0.670 | -8.027, -5.399 | 19.452 | 9.453 | 0.925, 37.980 |
| TS | WA | + | -0.035 | 0.002 | -0.038, -0.031 | -0.013 | 0.013 | -0.039, 0.013 |
| | TEMP | + | 1.529 | 0.054 | 1.424, 1.634 | 1.855 | 0.705 | 0.473, 3.237 |
| | MP | - | 0.053 | 0.004 | 0.044, 0.062 | 0.060 | 0.045 | -0.027, 0.147 |
| | SLOPE | - | -1.379 | 0.080 | -1.537, -1.221 | -0.683 | 0.642 | -1.940, 0.575 |
| | SLOPECV | - | 3.659 | 0.183 | 3.301, 4.018 | 0.751 | 1.508 | -2.205, 3.707 |
| | NDVI | NS | -13.333 | 0.595 | -14.50, -12.17 | 1.185 | 5.578 | -9.748, 12.118 |
| | NPP | NS | -0.009 | 0.002 | -0.012, -0.006 | -0.036 | 0.023 | -0.081, 0.010 |
| | NDVICV | NS | 12.189 | 0.633 | 10.957, 13.440 | 10.620 | 6.195 | -1.521, 22.761 |

**Table S4:** Name, location and protection status of wetlands using in our analysis.

| Lake Name | Province | County | Longitude | latitude | Protect status | Ramsar Site |
|---|---|---|---|---|---|---|
| Shengjin Hu | Anhui | Dongzhi | 30° 23′54″ | 117° 03′45″ | NNR | No |
| Caizi Hu | Anhui | Zongyang | 30° 48′36″ | 117° 05′48″ | PNR | No |
| Longgan Hu | Anhui | Susong | 29° 56′36″ | 116° 08′41″ | PNR | No |
| Huangda Hu | Anhui | Susong | 30° 01′26″ | 116° 20′34″ | PNR | No |
| Po Hu | Anhui | Taihu | 30° 09′19″ | 116° 27′28″ | PNR | No |
| Wuchang Hu | Anhui | Wangjiang | 30° 15′35″ | 116° 42′48″ | PNR | No |
| Baidang Hu | Anhui | Zongyang | 30 °48′16″ | 117° 22′25″ | PNR | No |
| Fengsha Hu | Anhui | Zongyang | 30° 55′37″ | 117° 37′50″ | PNR | No |
| Pogang Hu | Anhui | Yingjiang | 30° 39′05″ | 117° 10′10″ | PNR | No |
| Chang Hu | Hubei | Jinzhou | 30° 26′18″ | 112° 26′50″ | CNR | No |
| Liangzi Hu | Hubei | Liangzihu | 30° 16′43″ | 114° 34′46″ | PNR | No |
| Hong Hu | Hubei | Honghu | 29° 50′47″ | 113° 20′54″ | PNR | Yes |
| Wang Hu | Hubei | Xinyang | 29° 51′53″ | 115° 19′32″ | PNR | No |
| Shupo Hu | Hubei | Yangxin | 29° 49′56″ | 115° 23′26″ | NS | No |
| Maoli Hu | Hunan | Jinshi | 29° 24′00″ | 111° 57′37″ | NS | No |
| Beimin Hu | Hunan | Li | 29° 42′55″ | 111° 52′36″ | NS | No |
| Longchi Hu | Hunan | Hanshou | 28° 49′19″ | 112° 11′05″ | PNR | Yes |
| Anle Hu | Hunan | Hanshou | 28° 49′14″ | 112° 11′10″ | PNR | Yes |
| Muping Hu | Hunan | Hanshou | 28° 58′58″ | 112° 14′23″ | PNR | Yes |
| Wanzi Hu | Hunan | Yuanjiang | 28° 49′14″ | 112° 29′59″ | PNR | Yes |
| Datong Hu | Hunan | Yuanjiang | 29° 12′29″ | 112° 30′25″ | PNR | Yes |
| Lu Hu | Hunan | Yujiang | 29° 06′07″ | 112° 46′55″ | NNR | Yes |
| Hongqi Hu | Hunan | Yueyang | 29° 15′31″ | 112° 57′45″ | NNR | Yes |
| Fangtai Hu | Hunan | Yueyang | 29° 31′05″ | 112° 45′56″ | NNR | Yes |
| Chunfeng Hu | Hunan | Yueyang | 29° 13′25″ | 113° 03′43″ | NNR | Yes |
| Junshanhou Hu | Hunan | Yueyang | 29° 22′21″ | 113° 00′16″ | NNR | Yes |
| Dingzidiwai Hu | Hunan | Yueyang | 29° 26′00″ | 112° 55′04″ | NNR | Yes |
| Caisang Hu | Hunan | Yueyang | 29° 31′06″ | 112° 47′46″ | NNR | Yes |
| Daxiaoxi Hu | Hunan | Yueyang | 29° 29′19″ | 112° 48′12″ | NNR | Yes |
| Wangjun Hu | Hunan | Yueyang | 28° 51′41″ | 112° 33′38″ | NNR | Yes |
| Dong Hu | Hunan | Huarong | 30° 33′40″ | 114° 23′56″ | NS | No |
| Baini Hu | Hunan | Yunxi | 28° 44′59″ | 112° 52′29″ | NS | No |
| Helong Hu | Hunan | Xiangyin | 28° 41′00″ | 112° 49′55″ | NS | No |
| Beisai Hu | Hunan | Yueyang | 29° 07′20″ | 112° 59′53″ | NS | No |
| Hongze Hu | Jiangsu | Sihong | 33° 18′19″ | 118° 59′53″ | NNR | No |
| Baoying Hu | Jiangsu | Baoying | 33° 08′27″ | 119° 18′00″ | CNR | No |
| Gaoyou Hu | Jiangsu | Gaoyou | 32° 49′51″ | 119° 18′05″ | CNR | No |
| Dazong Hu | Jiangsu | Yandu | 33° 08′55″ | 119° 48′44″ | NS | No |
| Nan Hu | Jiangxi | De'an | 29° 12′00″ | 115° 49′35″ | CONR | Yes |
| Yangjia Hu | Jiangxi | Xingzi | 28° 50′00″ | 116° 49′35″ | CONR | Yes |
| Sixia Hu | Jiangxi | Xingzi | 29° 16′35″ | 115° 54′00″ | CONR | Yes |
| Shili Hu | Jiangxi | Xingzi | 29° 26′27″ | 116° 01′30″ | CONR | Yes |
| Liaohua Chi | Jiangxi | Xingzi | 29° 20′29″ | 115 °59′29″ | CONR | Yes |
| Chang Hu | Jiangxi | Xingxi | 29° 11′21″ | 115° 54′17″ | CONR | Yes |
| Xinmiao Hu | Jiangxi | Duchang | 29° 21′29″ | 116° 10′55″ | PNR | Yes |
| Duchangxi Hu | Jiangxi | Duchang | 29° 14′40″ | 116° 28′07″ | PNR | Yes |
| Shu Hu | Jiangxi | Duchang | 29° 11′30″ | 116° 20′33″ | PNR | Yes |
| Nanshan Hu | Jiangxi | Duchang | 29° 15′47″ | 116° 11′48″ | PNR | Yes |
| Jishan Hu | Jiangxi | Duchang | 29° 17′34″ | 116° 08′34″ | PNR | Yes |
| Gang Hu | Jiangxi | Hukou | 29° 41′24″ | 116° 13′45″ | CONR | Yes |
| Zao Hu | Jiangxi | Hukou | 29° 34′17″ | 116° 11′24″ | CONR | Yes |
| San Hu | Jiangxi | Nanchang | 28° 53′19″ | 116° 16′25″ | CONR | Yes |
| Saicheng Hu | Jiangxi | Jiujiang | 29° 41′52″ | 115° 52′05″ | CONR | Yes |
| Nan Hu | Jiangxi | Yugan | 28° 49′07″ | 116° 15′21″ | PNR | Yes |
| Linchong Hu | Jiangxi | Yugan | 28° 51′14″ | 116° 16′16″ | PNR | Yes |
| Xi Hu | Jiangxi | Yugan | 28° 50′53″ | 116° 14′00″ | PNR | Yes |
| Chengjia Hu | Jiangxi | Yugan | 28° 48′08″ | 116° 18′11″ | PNR | Yes |
| Caowan Hu | Jiangxi | Yugan | 28° 50′50″ | 116° 19′07″ | PNR | Yes |
| KangshanNei Hu | Jiangxi | Yugan | 28° 51′41″ | 116° 29′15″ | PNR | Yes |
| Qingshan Hu | Jiangxi | Poyang | 29° 07′34″ | 116° 38′47″ | NS | No |
| Chang Hu | Jiangxi | Xinjian | 28° 55′32″ | 116° 17′22″ | NNR | Yes |
| Sanniwan Hu | Jiangxi | Xinjian | 28° 54′11″ | 116° 18′58″ | NNR | Yes |
| Zhanbei Hu | Jiangxi | Xinjian | 28° 54′50″ | 116° 16′17″ | NNR | Yes |
| Shi Hu | Jiangxi | Xinjian | 28° 52′39″ | 116° 18′24″ | NNR | Yes |

| | | | | | | |
|---|---|---|---|---|---|---|
| Dawu Hu | Jiangxi | Xinjian | 29 °01′14″ | 116° 09′06″ | NNR | Yes |
| Qinglan Hu | Jiangxi | Jinxian | 28° 28′00″ | 116° 10′08″ | PNR | Yes |
| Mingxi Hu | Jiangxi | Xinjian | 28° 58′28″ | 116° 14′06″ | NS | No |
| Candou Hu | Jiangxi | Xinjian | 29° 05′55″ | 116° 05′15″ | NNR | Yes |
| Xiaotan Hu | Jiangxi | Xinjian | 29° 04′02″ | 116° 07′04″ | NNR | Yes |
| Dahu Chi | Jiangxi | Yongxiu | 29° 07′42″ | 115° 56′43″ | NNR | Yes |
| Zhushi Hu | Jiangxi | Yongxiu | 29° 10′36″ | 115° 58′20″ | NNR | Yes |
| Changhu Chi | Jiangxi | Yongxiu | 29° 08′16″ | 115° 59′19″ | NNR | Yes |
| Beng Hu | Jiangxi | Xingzi | 29° 13′48″ | 115° 57′27″ | NNR | Yes |
| Sha Hu | Jiangxi | Xingzi | 29° 10′51″ | 115° 55′50″ | NNR | Yes |
| Dacha Hu | Jiangxi | Yongxiu | 29° 09′27″ | 116° 05′07″ | NNR | Yes |
| Zhonghu Chi | Jiangxi | Xinjian | 29° 09′56″ | 116° 01′05″ | NNR | Yes |
| Meixi Hu | Jiangxi | Yongxiu | 29° 13′11″ | 116° 03′26″ | NNR | Yes |
| Xiang Hu | Jiangxi | Xinjian | 29 °06′56″ | 116° 00′34″ | NNR | Yes |

NNR=National nature reserve

PNR=Provincial nature reserve

CNR=City nature reserve

CONR=County nature reserve

NS = Not protect area

**Table S5:** Time-series census data using in our analysis in four key sites in the Yangtze.

| Lake name | Reserve | 2000/01 | 2001/02 | 2002/03 | 2003/04 | 2004/05 | 2005/06 | 2006/07 | 2007/08 | 2008/09 | 2009/10 | 2010/2011 | 2011/2012 |
|---|---|---|---|---|---|---|---|---|---|---|---|---|---|
| Shengjin Hu | Shengjin | NS | NS | NS | Feb[1] | Feb[2] | Feb[3] | Feb[3] | Feb[7] | Feb[7] | Feb[7] | Feb[7] | Feb[7] |
| Wuchang Hu | Anqing | NS | NS | NS | Feb[1] | Feb[2] | Mar[4] | Jan[4] | Jan[4] | Dec[7] | Feb[7] | Feb[7] | Dec[7] |
| Caizi Hu | Anqing | NS | NS | NS | Feb[1] | Feb[2] | Mar4 | Jan[4] | Jan[4] | Dec[7] | Feb[7] | Feb[7] | Dec[7] |
| Baidang Hu | Anqing | NS | NS | NS | Feb[1] | Feb[2] | NS | Jan[4] | Feb[4] | Dec[7] | Feb[7] | Feb[7] | Dec[7] |
| Fengsha Hu | Anqing | NS | NS | NS | Feb[1] | Feb[2] | Mar[4] | Jan[4] | Feb[7] | Dec[7] | Feb[7] | Feb[7] | Dec[7] |
| Huang Hu | Anqing | NS | NS | NS | Feb[1] | Feb[2] | Mar[4] | Jan[4] | Jan[4] | Dec[7] | Feb[7] | Feb[7] | Dec[7] |
| Po Hu | Anqing | NS | NS | NS | Feb[1] | Feb[2] | Mar[4] | Jan[4] | Jan[4] | Jan[7] | Feb[7] | Feb[7] | Dec[7] |
| Nan Hu | Nan hu | Jan[5] | Jan[5] | Jan[5] | Feb[1] | Feb[2] | Dec[5] | Dec[5] | Jan[5] | Feb[5] | Feb[5] | Feb[5] | Feb[5] |
| Shili Hu | Xing zi | Jan[5] | Jan[5] | Jan[5] | Feb[1] | Feb[2] | Dec[5] | Dec[5] | Jan[5] | Feb[5] | Feb[5] | Feb[5] | Feb[5] |
| Liaohua Chi | Xing zi | Jan[5] | Jan[5] | Jan[5] | Feb[1] | Feb[2] | Dec[5] | Dec[5] | Jan5 | Feb5 | Feb5 | Feb5 | Feb5 |
| Chang Hu | Xing zi | Jan[5] | Jan[5] | Jan[5] | Feb[1] | Feb[2] | Dec[5] | Dec[5] | Jan[5] | Feb[5] | Feb[5] | Feb[5] | Feb[5] |
| Xingmiao Hu | Du chang | Jan[5] | Jan[5] | Jan[5] | Feb[1] | Feb[2] | Dec[5] | Dec[5] | Jan[5] | Feb[5] | Feb[5] | Feb[5] | Feb[5] |
| Duchangxi Hu | Du chang | Jan[5] | Jan[5] | Jan[5] | Feb[1] | Feb[2] | Dec[5] | Dec[5] | Jan[5] | Feb[5] | Feb[5] | Feb[5] | NS |
| Jishan Hu | Du chang | Jan[5] | Jan[5] | Jan[5] | Feb[1] | Feb[2] | Dec[5] | Dec[5] | Jan[5] | Feb[5] | Feb[5] | Feb[5] | NS |
| Gang Hu | Ping feng | Jan[5] | Jan[5] | Jan[5] | Feb[1] | Feb[2] | Dec[5] | Dec[5] | Jan[5] | Feb[5] | Feb[5] | Feb[5] | Feb[5] |
| Zao Hu | Ping feng | Jan[5] | Jan[5] | Jan[5] | Feb[1] | Feb[2] | Dec[5] | Dec[5] | Jan[5] | Feb[5] | Feb[5] | Feb[5] | Feb[5] |
| Jiangxiangsan Hu | Shan hu | Jan[5] | Jan[5] | Jan[5] | Feb[1] | Feb[2] | Dec[5] | Dec[5] | Jan[5] | Feb[5] | Feb[5] | Feb[5] | NS |
| Saicheng Hu | Sai cheng | Jan[5] | Jan[5] | Jan[5] | Feb[1] | Feb[2] | Dec[5] | Dec[5] | Jan[5] | Feb[5] | Feb[5] | Feb[5] | Feb[5] |
| Linchong Hu | Yu gan | Jan[5] | Jan[5] | Jan[5] | Feb[1] | Feb[2] | Dec[5] | Dec[5] | Jan[5] | Feb[5] | Feb[5] | Feb[5] | Feb[5] |
| Chang Hu | Nanji shan | Jan[5] | Jan[5] | Jan[5] | Feb[1] | Feb[2] | Dec[5] | Dec[5] | Jan[5] | Feb[5] | Feb[5] | NS | Feb[5] |
| Sanniwan Hu | Nanji shan | Jan[5] | Jan[5] | Jan[5] | Feb[1] | Feb[2] | Dec[5] | Dec[5] | Jan[5] | Feb[5] | Feb[5] | Feb[5] | Feb[5] |
| Dawu Hu | Nanji shan | Jan[5] | Jan[5] | Jan[5] | Feb[1] | Feb[2] | Dec[5] | Dec[5] | Jan[5] | Feb[5] | Feb[5] | Feb[5] | Feb[5] |
| Qinglan Hu | Jin xian | Jan[5] | Jan[5] | Jan[5] | Feb[1] | Feb[2] | Dec[5] | Dec[5] | Jan[5] | Feb[5] | Feb[5] | Feb[5] | Feb[5] |
| Poyang Hu NNR | Po yang | Jan[5] | Jan[5] | Jan[5] | Feb[1] | Feb[2] | Dec[5] | Dec[5] | Jan[5] | Feb[5] | Feb[5] | Feb[5] | Feb[5] |
| East Dongting NNR | East Dongting | NS | NS | NS | Feb[1] | Feb[2] | Jan[6] | Jan[6] | Feb[6] | Feb[7] | Feb[7] | Feb[7] | Jan[6] |

1 = WWF 2004 middle-lower Yangtze River floodplain survey; 2 = WWF 2004 middle-lower Yangtze River floodplain survey

3 = Shengjin Lake National Nature Reserve survey; 4 = Anqing Yanjiang Nature Reserve survey

5 = Poyang Lake National Reserve survey; 6 = Dongdongting Lake National Nature Reserve survey

7 = Survey conducted by authors; NS = No data available

**Table S6:** Information of satellite images used in this study.

| Date | Path/Row | Sensor | Cloud cover (%) |
|---|---|---|---|
| 2004-01-24 | 119/038 | ETM+ | 0 |
| 2004-01-27 | 124/039 | ETM+ | 24 |
| 2004-01-27 | 124/040 | ETM+ | 16 |
| 2004-02-16 | 120/039 | ETM+ | 0 |
| 2004-01-21 | 122/039 | TM | 0 |
| 2004-02-08 | 120/037 | TM | 23 |
| 2004-02-08 | 120/038 | TM | 0 |
| 2004-02-13 | 123/039 | TM | 0 |
| 2004-02-13 | 123/040 | TM | 0 |
| 2004-02-15 | 121/039 | TM | 0 |
| 2004-02-15 | 121/040 | TM | 0 |

**Table S7:** Pearson Correlation coefficients between independent variables, and the Variance Inflation Factors (VIF); n = 78; (*$P < 0.05$;**$p < 0.01$;***$p < 0.001$). For abbreviations see table 4.

| | LA | WA | TEMP | MP | NDVI | NPP | SLOPE | SLOPECV | NDVICV | VIF |
|---|---|---|---|---|---|---|---|---|---|---|
| LA | | | | | | | | | | 1.914 |
| WA | 0.165 | | | | | | | | | 3.290 |
| TEMP | -0.111 | -0.492** | | | | | | | | 2.014 |
| MP | 0.249* | -0.115 | 0.035 | | | | | | | 2.275 |
| NDVI | -0.021 | -0.184 | 0.303** | -0.067 | | | | | | 1.750 |
| NPP | 0.152 | -0.012 | -0.018 | 0.080 | -0.034 | | | | | 1.923 |
| SLOPE | 0.019 | 0.000 | 0.107 | 0.235* | 0.024 | 0.072 | | | | 1.745 |
| SLOPECV | 0.084 | -0.013 | -0.031 | 0.246* | -0.018 | 0.068 | 0.450** | | | 1.686 |
| NDVICV | 0.285* | -0.021 | -0.158 | 0.086 | -0.027 | 0.206 | 0.024 | 0.110 | | 2.275 |

**Table S8:** Moran's I values of residuals for the test of spatial autocorrelation for each species.

| Species | Moran's I | *p* |
|---|---|---|
| Bean goose | 0.020 | 0.279 |
| Greater white-fronted goose | 0.003 | 0.618 |
| Lesser white-fronted goose | 0.027 | 0.137 |
| Swan goose | 0.021 | 0.220 |
| Tundra swan | -0.030 | 0.661 |

**Appendix S1**

**Satellite image processing**

We used 11 Landsat images from January to February of 2004 (coinciding with the survey date) to cover the temporal and spatial scale of our study (Table S3).

After 2003, ETM+ images contain data duplications and loss due to the failure of the Scan Line Corrector[1]. We therefore involved a gap-filling method based on local linear histogram matching[2] for the four ETM+ images. Digital Number (DN) values were then calibrated to radiations before FLAASH (Fast Line-of-sight Atmospheric Analysis of Hypercubes). All Images were geometrically registered and resampled with a cubic convolution algorithm.

We firstly registered the 2004 image (path/row 121/039) as the base image. Then Radiometric Normalization for Image Mosaics (RNIM) was conducted using the registered image as the new master image to normalize the other ones[3,4] (Du et al. 2001, Olthof et al. 2004). RNIM applies Principles Component Analysis (PCA) in overlap regions, with the first principal component performing a least-squares regression between overlap regions and the second principal component detecting changing elements between two scenes. By adjusting the ranges of the second principal component, we removed changed pixels until a minimum correlation coefficient of 0.9 was reached (Du et al. 2001, Olthof et al. 2004). RNIM has the unique advantage that it generates similar results regardless of the different processing orders (Du et al. 2001).

We delineated boundaries for all 78 lakes in our study area through Google Earth

and field GPS records as a mask for classification. We used Supported Vector Machines (SVMs) to discriminate water and land for each lake. As pixels containing water or land are quite contrasting, we selected our training data visually from each image based on a few field survey records. Using ArcGIS 10.0, land area, average NDVI of recessional grassland and standard deviation this NDVI value were calculated for each lake with Zonal statistic toolkit.

**Reference**

1 USGS. (2013) Available at: http://landsat.usgs.gov/products_slcoffbackground.php. (Accessed: 21st May 2014)

2 Scaramuzza, P., Micijevic, E. & Chander, G. SLC Gap-filled products phase one methodology. (2004) Available at: http://landsat.usgs.gov/documents/SLC_Gap_Fill_Methodology.pdf. (Accessed: 28th May 2014)

3 Du Y., Cihlar J., Beaubien J. & Latifovic R. Radiometric normalization, compositing, and quality control for satellite high resolution image mosaics over large areas. *IEEE Trans. Geosci. Remote Sens.* **39,** 623-634 (2001).

4 Olthof I., Pouliot D., Fernandes R. & Latifovic R. Landsat-7 ETM+ radiometric normalization comparison for northern mapping applications. *Remote Sens. Environ* **95,** 388-398 (2004).
